# 

**Published:** 1897-01

**Authors:** 


### INDEX TO VOLUME XXVII.

PAGE

Anatomy of Rape. The ... 76
Acute Lobar Pneumonia ... 1C4
Associated Study of Crime and Insanity ... 132
Albumin in the Urine ... 128
Action, Application and Value of Oleum Eucalypti (Sander) ... 185
Acute Tuberculosis of Larynx ... 214
Appendicitis, With Report of Twenty-Six Cases ... 226
Abscess of Both Kidneys Due to Chronic Cystitis ... 325
Atlanta Society of Medicine, 432, 379, 354, 383
American Medical Association ... 352
Atlanta and the Yellow Jack ... 520
Abstract of a Letter from Dr Sajous ... 522

Book Reviews ... 574, 575, 411, 254
Boiled Milk ... 307
Bacillus of the Plague ... 240
Bullets in the Brain and the Roentgen Rays ... 176
Beef Essence ... 188
Blennorrhœa Treated with Ichthyol ... 294

Contribution to the Therapeutics of Iron, A ... 546
Condensed Milk as an Infant Food ... 561
Common Sense in the Use of Antiseptics 517
Calcareous Uterine Fibroid ... 484
Clinical Report on Pepto-Mangan ... 487
Crepitant Rale an Interpleural Sound, The ... 373
Chronic Malarial Toxemia ... 3[illegible]6
Castration for Rape in Kansas ... 399
Competition for the Tennessee Medal ... 405
Chronic Specific Urethritis ... 488
Chronic Otorrhœa, Treatment of ... 265
Chronic Interstitial Nephritis in Childhood ... 284
Care of the Teeth in Children ... 286
Case of Castration for Hypertrophied Prostate ... 306
Congestion of Uterus, Its Causes and Treatment ... 155
Condensed Facts Concerning Electricity 173
Curious Case of Religious Monomania ... 189
Contagious Impetigo ... 189
Comparative Value of Eucaine and Cocaine, The ... 190
Cistern, The, a Substitute for the Country Well ... 193
Coitus as a Disturbing Element ... 116
Calcium Sulphydrate, Depilatory ... 118

PAGE

Correspondence ... 37, 142
Catching Cold ... 85
Cosmetics of Castration ... 85
Cocaine Poisoning, Magnan's Symptoms 95
Clinical Study of Nasal Syphilis ... 1
Croupous Dysentery, Case of ... 11

Demand and Supply of Eunuchs in China, The ... 82
Dacryocystitis and Its Treatment ... 86
Diabetes in a Young Child ... 137
Diagnosis of Carcinoma of Breast in Early Stages ... 123
Diagnosis and Treatment of Lumbago, The ... 175
Dangers of Morphine ... 181
Diet in Dyspepsia ... 242
Degree of Anæsthesia Which Should Be Induced Prior to Surgical Operations, The ... 300
Doctors' Bills Must be Paid ... 299
Disguises of Fever, The ... 350
Death by Lightning ... 330
Doctors of Osteopathy ... 452
Danger of Vaginal Injection, The ... 457
Demonstration of the Pfeiffer-Widal Typhoid Serum Reaction ... 465
Death of Father Kneipp ... 406
Differential Diagnosis Between Malarial and Typhoid Fevers ... 512
Distinguished Physician-Pharmacist ... 537
Dangers of Treating Gonorrhœa with Simple Astringents ... 593
Dietetic Treatment of Diabetes, The ... 622

Editorial, 627, 571, 520, 406, 468, 363, 310, 252, 195 140, 95, 32 ...
Ether-Chloroform Controversy, The ... 494
Examination of Throats of Children ... 511
Euthanasia ... 408
Earache ... 275
Emile Zola as a Degenerate ... 180
Etiology of Acute Rheumatism ... 138
Epithelial Sowing ... 90
Early Signs of Locomotor Ataxia ... 78

Feeding of the Sick, The ... 83
Few Notes on Varix, etc., A ... 160
Facial Eczema ... 345
Five Successful Cases of General Suppurative Peritonitis ... 459
Fashions and Fancies in Food ... 402
Fracture of the Penis ... 508
Football ... 571

PAGE

Gaston, J. McF., Remarks on Opening Course in Surgery, Southern Medical College .......... 531
Gunshot Wounds and Their Treatment .. 489
Glandular Fevers of Childhood, The ..... 392

Hydrozone in Gastric and Intestinal Disorders .......... 80
Hypertrophic Elongation of Cervix Uteri etc .......... 53
Hernia .......... 183
Horsley's Antiseptic Wax .......... 249
Hysterectomy for Uterine Fibroids ...... 217
Hydrastic Canadensis in Bronchial Catarrh .......... 295
Helminthiasis Extraordinary .......... 296

Iron and Manganese Peptonate .......... 620
Irregular Menstruation in Young Women 611
Identification of the Dead, The .......... 562
Indifference of Patients .......... 497
Ingrown Toe-nails .......... 518
Infantile Diarrhœa .......... 398
Inflammation of the Skin as a Result of the Poison of Plants .......... 446
Is Malaria a Water-borne Disease .......... 448
Indigestion of Breast-fed Babies, The ... 341
Insane Kings .......... 246
Inheritance of Mutilations, etc. .......... 250
Interaction of Somatic and Psychic Disorders .......... 125
Icebag in Pneumonia, The .......... 136
Indications for Ventral Suspension of Uterus .......... 88

Janet Abortive Treatment of Gonorrhœa 73

Kinks in Urine Examination .......... 225

Laryngeal Diphtheria .......... 321
Liability for Maliciously Filing Information Charging Insanity .......... 401
Lime Water as a Disinfectant for Linen and Cotton Fabrics .......... 401
Ligation of Dorsal Vein of Penis for Impotency .......... 616

Mahar Quarter, A .......... 559
Modern Treatment of Typhoid .......... 79
Massage in Treatment of Muscular Rheumatism .......... 92
Medical Treatment of Inebriety .......... 134
Microscopic Note .......... 181
Morphine-Ether Narcosis .......... 182
Multiple Bilateral Cystoma of the Organ of Giraldes .......... 171
Milk as a Source of Infection .......... 220
Meeting of the Georgia Medical Association .......... 252
Merycism .......... 287
Modern Technic of Cæsarian Section .... 618

PAGE

New Method of Localizing Brain Lesions 305
Newspaper Attack on the Code of Ethics 301
Notes on Treatment of Fæcal Fistula .... 87
Nerve Element in Surgical Pathology, The .......... 371
Ninth Annual Meeting Tri-State Medical Society .......... 514
New Method of Inviting Sleep, A .......... 509
New Test for Typhoid Fever, A .......... 614

Operative Treatment of Facial Neuralgia 400
Our Immunity from Plague .......... 113
Operative Treatment of Irreducible Dislocation of Shoulder .......... 504
Ovarian Abscess .......... 377

Pathology of Recurrent Appendicitis .... 443
Pathology of Diabetes Insipidus .......... 464
Pathognomonic Sign of Acute Miliary Tuberculosis of Lungs, A .......... 32[illegible]
Prevention and Treatment of Insanity of Pregnancy and Puerperal State .......... 244
Phosphuria in Diagnosis .......... 249
Puerperal Eclampsia .......... 112
Pregnancy Diagnosed by Urine .......... 133
Primary Lateral Sclerosis .......... 58
Proper Management of Appendicitis .... 91
Peculiar Virtues of Cod-liver Oil .......... [illegible]3
Poultices in Pulmonary Diseases of Children .......... 558

Question of Treating the Poor Who Can Not Pay the Physician, The .......... 393

Round Shoulders in Girls .......... 564
Recurrent Appendicitis .......... 536
Report of Case Croupous Dysentery ..... 11
Retinal Hemorrhage Caused by Lithemia 65
Renipuncture in Albuminuria .......... 184
Report of a Peculiar Case .......... 209
Remarks on Appendicitis .......... 226
Radical Operation for Varicose Veins ... 258
Remedy in Nervous Disorders, Etc., A. .. 3[illegible]6
Richmond Academy of Medicine and Surgery .......... 308
Recent Advances in General Medicine ... 290
Reciprocity in Medical Licensure .......... 361
Remedies that Enslave .......... 4[illegible]0
Role of Bacillus of Typhoid Fever ....... 468
Successful Treatment of Sarcoma, etc ... 423
Suprapubic Cystotomy for Stone .......... 454
So-called Ozomulsion .......... 343
Study of American Medicinal Flora ...... 347
Some Evils that Threaten the Medical Profession .......... 271
Sage in Hyperidrosis .......... 283
Significance of Sudden Acute Abdominal Pain .......... 292
Simple Method of Sterilizing Rubber Catheters .......... 297
Supposed Precocious Menstruation ...... 251

PAGE
Surgery of Country Practitioner, The.... 245
Some Dottings on Hemorrhage.......... 170
Skiagraphs in Diagnosing Thoracic Diseases.................................. 192
Some Observations on Medical Topics in Fiction .................................. 120
Sea Water and Menstruation ............. 123
Suture—Clamp Operation for Hemorrhoids....................................... 89
Surgery of Appendix........................ 74
Simple Method of Treating Opium Poisoning ..................................... 76
Surgical hints ........................... 77
Short Lecture on Influenza................ 68
Southern Surgical and Gynecological Association .................................. 13
Sub-peritoneal Myoma...................... 389
Some Water Uses Well to Remember.. 397
Summer Complaints—Prophylaxis....... 4 3
Sudden Death ................................ [illegible]65
Swear off ........................................ 571
Strangulated Hernia......................... 585
Surgical Uses of Picric Acid............. 595
Simple Method of Removing Foreign Bodies From Nasal Cavities of Children............................................ 615
Specialists and Specialties................ 623

That Wave of Prosperi y................... 519
Treatment of Insect Bites.................. 621
Treatment of Cerebral Hemorrhage .. 606
Treatment of Syphilis....................... 610
Tenotomy in Convergent Squint......... 583
Treatment of Carcinoma of Uterus...... 568
Tobacco Habit Among the Young, The.. 572
Treatment of Caries of Spine.... ....... 552

PAGE
Traumatic (?) Stricture of Rectum...... 477
Treatment of Detachment of the Retina 93
Town Enslaved by the Côcaine Habit.... 135
Treatment of Ununited Fractures........ 101
Tubercular Meningitis........................ 107
Treatment of Potts' Curvature........... 188
Treatment of Placenta Previa............. 195
Treatment of Fracture........................ 241
Twelfth International Medical Congress 253
Total Abdominal Hysterectomy........... 263
Treatment of Varicose Veins.............. 282
Treatment of Dysmenorrhœa with Galvanism.......................................... 291
Tonsillar Cough................................ 299

Uncommon Affections of Eyelids ....... 177
Unconsciousness from Constipation..... 179
Use of Alcohol in Sudden Illness........ 29
Uses and Limitations of Condensed Milk as Baby Food..................................... 561

Various Discharges of the Male Urethra 280
Venomous Snakes (a request)............ 623
Value to Public of State Medical Societies............................................. 499
Venereal Buboes ............................... 133
Vaginal Ligation of Uterine Arteries for Fibromata......................................... 136
Vinegar as a Hemostatic.................... 242

Wonders of Modern Medicine and Surgery............................................. 566
Wound of the Heart, Recovery............ 560
Woman's Place in Medicine................ 394
Water Treatment For Atrophy............ 340
What Can the Country Doctor do to Prevent Typhoid Fever............................ 339
Yellow Fever in the Olden Time.......... 566

